# Quantitative Immunohistochemical Analysis Reveals Association between Sodium Iodide Symporter and Estrogen Receptor Expression in Breast Cancer

**DOI:** 10.1371/journal.pone.0054055

**Published:** 2013-01-14

**Authors:** Sushmita Chatterjee, Renu Malhotra, Frency Varghese, Amirali B. Bukhari, Asawari Patil, Ashwini Budrukkar, Vani Parmar, Sudeep Gupta, Abhijit De

**Affiliations:** 1 Functional Molecular Imaging Lab, ACTREC, Tata Memorial Centre, Kharghar, Navi Mumbai, India; 2 Molecular Pathology, Tata Memorial Hospital, Parel, Mumbai, India; 3 Department of Radiation Oncology, Tata Memorial Hospital, Parel, Mumbai, India; 4 Department of Surgery, Tata Memorial Hospital, Parel, Mumbai, India; 5 Department of Medical Oncology, Tata Memorial Hospital, Parel, Mumbai, India; University of Medicine and Dentistry of New Jersey, United States of America

## Abstract

**Background:**

Human sodium iodide symporter (hNIS) gene over-expression is under active consideration worldwide as an alternative target molecule for breast cancer (BC) diagnosis and targeted radio-iodine treatment. However, the field demands better stratified analysis of endogenous hNIS expression across major BC subtypes. Therefore, we have analyzed subtype-specific variation of hNIS overexpression in breast tumor tissue samples by immunohistochemistry (IHC) and also report the development of a homogeneous, quantitative analysis method of digital IHC images.

**Methods:**

hNIS expression was analyzed from 108 BC tissue samples by IHC. Sub-cellular localization of hNIS protein was analyzed by dual immunofluorescence (IF) staining method using hNIS and HER2 antibodies. An ImageJ based two-step digital analysis method was developed and applied for the bias-free analysis of the images.

**Results:**

Staining of the tumor samples show 70% cases are hNIS positive indicating high incidence of hNIS positive cases in BC. More importantly, a subtype specific analysis done for the first time shows that hNIS expression is overly dominated in estrogen receptor (ER) positive cases than the receptor negative cases. Further, 56% of the ER+ve, PgR+ve, HER2-ve and 36% of ER+ve, PgR+ve, HER2+ve cases show highest intensity staining equivalent to the thyroid tissue. A significant positive correlation is also observed between hNIS and estrogen receptor expression (p = 0.0033, CI = 95%) suggesting hNIS mediated targeted radio-iodine therapy procedures may benefit both ER+ve, PgR+ve, HER2–ve as well as HER2+ve cases. Further, in a few cases, hNIS and HER2 protein localization is demonstrated by overlapping membrane co-expression. ImageJ based image analysis method shows over 70% match with manual pathological scoring method.

**Conclusion:**

The study indicates a positive link between hNIS and ER expression in BC. The quantitative IHC image analysis method reported here will further help in patient stratification and potentially benefit global clinical assessment where hNIS mediated targeted ^131^I radio-ablative therapy is aimed.

## Introduction

Since, the human sodium iodide symporter (hNIS) cDNA was cloned [1], attempts to examine its expression in various human non-thyroidal cell lines and correlate with its function as a membrane iodine transporter has been initiated. Harboring the natural overexpression of hNIS targeted radio-iodine treatment of patients are routinely being used in thyroid clinics [2], [3]. In a pioneering study, Tazebay *et al*. demonstrated that above 80% of invasive and ductal carcinoma *in situ* (DCIS) samples express hNIS, whereas only 20% of tumor adjacent normal tissues express this protein [4]. Wapnir *et al*. analyzed hNIS expression in a total of 202 human breast samples and reported hNIS positivity in 76% of invasive, 88% of DCIS (Ductal carcinoma in situ) and 80% of fibroadenoma samples [5]. Over the last ten years, various groups around the world have reported hNIS over-expression in breast tumor tissue samples [6]–[12]. Additionally, it is also evident from literature that hNIS can be effectively used as a functional reporter protein in non-thyroidal cells [13]–[17]. All these evidences together suggest that 80% cases are positive compared to none in non-lactating normal breast and show promises in utilizing the over-expressing hNIS protein in breast cancer. The hNIS mediated iodide accumulation was also evaluated in patient volunteers with metastatic breast cancer by scintigraphic method [12]. A recent review also cites clinical studies where endogenous hNIS over-expression in patient tumors has been successfully used to some extent [18]. As receptor negative breast cancer (e.g. ER-ve, PgR-ve, HER2–ve subtype) with worst prognosis lacks any targeted therapy option, studies across the world mostly focused at hNIS based radio-iodine treatment regimen in ER-ve, PgR-ve, HER2–ve subtype [6]–[10].

Another important consideration appeared later when Beyer et al. who has analyzed hNIS over-expression in patient tissue samples with a particular focus on sub-cellular localization of hNIS protein [19]. According to their report only 27% of hNIS positive tumors show that the symporter protein localizes at the cell surface, whereas in the remaining samples hNIS appears as cytoplasmic staining. This is in fact a very important observation that may explain the conflicting reports in literature where in spite of a very high percentage of hNIS positivity reported, only 17–25% of radionuclide uptake in BC patients is seen [20]. Yet, it was argued in literature whether immunohistochemical staining techniques and visual evaluation of stained tissue sections used as main analysis criteria could be taken as sufficient to comment on sub-cellular localization of a membrane protein [21]. Therefore requirement of an alternative staining technique and a universal quantitative method of measuring hNIS gene expression in tumor tissue samples are realized.

Among the various pathological procedures, immunohistochemistry (IHC) plays an important role that requires staining tumor tissue samples with antibodies specific to the molecular targets. Further, quantification of the immunostained sample (commonly called scoring) is often required to establish a judgment parameter in clinical decision making processes especially for cases where a targeted treatment is available against the marker. Nonetheless, due to high degree of biological variations in clinical samples, overall the scoring process is manual which relies on training human vision to the IHC color development and thus remain prone to inter- and intra-observer variability [22]. Despite of being such an important decision making step, the interpretation of such results is subjective and often causes inconsistencies in the evaluation process across the research laboratories. A classic example is the HER2 (Human epidermal growth factor receptor type 2) scoring in breast oncology where numerous research literatures have critically evaluated the process to come to a consensus [23], [24]. In order to make immunohistochemical studies more objective, automated quantitative techniques based on computer-assisted microscopy have been developed [23], [25]. The implementation of digital or computational pathology in routine tissue-based diagnosis has started, but is limited due to cost involvement and biological variability. Though many commercial IHC image analysis instruments are available now, affordability of such instruments remained as a major obstacle for research organizations or city hospitals. Overall, except for a few well established biomarkers, it’s still an ongoing process of standardizing clinical methodologies to meet the patients need worldwide.

Therefore during this study, we conducted a thorough analysis of a large dataset of 108 patient tumor tissue samples representing all major subtypes of BC and find that hNIS over-expression is significantly associated with estrogen receptor (ER) positive cases. We for the first time report development of a two-step semi-automated scoring methodology using an open resource digital image analysis program (ImageJ) and demonstrate its utility in quantitative, bias-free analysis of hNIS over-expression in breast tumor samples.

## Materials and Methods

### Ethics Statement

The clinical study protocol was reviewed and approved by the TMC-ACTREC Institutional Review Board. For all experiments paraffin embedded tissue blocks from our tumor tissue repository were used and thus patient consent waiver was obtained.

### Study Population

The paraffin embedded breast tumor tissue blocks were collected and used for hNIS immunostaining during this study. A total of 108 randomly collected tissue samples from patients between the age group of 32–72 years were obtained from ACTREC tumor tissue repository collected during the years 2008–2009. All tumor samples were confirmed as infiltrating duct carcinoma, grade III and were classified as 25 ER+ve, PgR +ve, HER2–ve, 11 ER+ve, PgR+ve, HER2+ve, 27 ER-ve, PgR–ve, HER2+ve, and 45 ER-ve, PgR-ve, HER2-ve samples on the basis of available standard immunohistochemistry report. Out of 108, 16 samples were obtained from the patients who had received preoperative chemotherapy treatment. Among the post chemotherapy cases, number of samples from different subtypes includes 4 ER+ve, PgR+ve, HER2-ve, 2 ER+ve, PgR+ve, HER2+ve, 3 ER-ve, PgR-ve, HER2+ve and 7 ER-ve, PgR-ve and HER2-ve. Of all cases, 48 patients were premenopausal (up to age 50 years) and 60 were postmenopausal (above 50 years). The demographic characters of patient cohort are mentioned in Table 1.

**Table 1:** Characteristic of patient cohort.

Total patients	108
***Age***	32–72 yrs
***Stage***	III
***Histology***	Infiltrating ductal carcinoma
***Chemotherapy status***	
Pre chemotherapy	92
Post chemotherapy	16
***Estrogen receptor***	
Positive	36
Negative	72
***Progesterone receptor***	
Positive	36
Negative	72
***HER2***	
Positive	38
Negative	70

### Immunohistochemistry and Immunofluorescence

Standard IHC protocol was followed to stain the tumor tissue samples using the mouse monoclonal antibody against hNIS (Abcam, Cat # ab17795). In brief, 5 µm sized paraffin embedded tissue sections were de-paraffinized with xylene and endogenous peroxidase activity was quenched with 3% H_2_O_2_ in methanol for 30 minutes in dark. Tissue sections were dehydrated through graded alcohols and subjected to antigen retrieval using 10 mM sodium citrate. Sections were washed with TBST (Tris borate saline-tween20) and then blocked with 5% BSA (Bovine serum albumine) for one hour. Slides were incubated with the mouse monoclonal antibody against hNIS diluted with TBS in 1∶50 ratio. Slides were washed for 5 minutes in TBST and incubated for 1 hour with HRP (Horse raddish peroxidase) conjugated anti mouse antibody diluted with TBS in 1∶200 ratio. After washing, slides were incubated with DAB (3,3′-diaminobenzidine tetrahydrochloride ) (Sigma) and immediately washed under tap water after the color development. Slides not incubated with anti hNIS antibody were used as secondary control (data not shown). For each batch of sample staining, a papillary thyroid carcinoma case was kept as positive control for hNIS expression. A salivary gland section was also used as positive control. All the slides were counter stained with haematoxylin. Slides were DPX mounted and observed under light microscope (Carl Zeiss).

For the purpose of immunofluorescence study, tissue sections were treated in the same way as for IHC. After blocking with BSA, sections were incubated with hNIS and HER2 (Abcam, ab8054) antibodies one by one and then stained with respective fluorescent dye labeled secondary antibodies. For hNIS staining Alexafluor 488 labeled rabbit secondary antibody was used where as HER2 was counter-stained with Dylight633 labeled goat secondary antibody. Antibody stained sections were washed with TBST and then mounted with vecta-shield (Vector Laboratories) and images were captured using a confocal microscope (Carl Zeiss).

### Pathological Scoring

Antibody stained permanent slides were examined under light microscope and sections showing less than 10% of stained area were considered as negative where as sections showing 10–20% of stained area were considered as focal, 20–70% of stained area were considered as patchy and more than 70–80% of stained area were considered as diffused. Intensity variation of the staining was visibly examined and scored by pathologist as 0, 1+ and 2+.

### Digital Image Analysis

IHC digital images were used for developing semi-automated analysis protocol. As a first step, we used a color de-convolution technique to un-mix the pure DAB, hematoxylin stained areas leaving a complimentary image. The pixel intensities of separated DAB or hematoxylin images range from 0 to 255. Value 0 represents the darkest shade of the color while 255 represent the lightest shade of the color in the image. In-order to assign an automated score by judging the pure DAB staining pattern, histogram profile of every image i.e. the number of pixels of a specific intensity value vs. their respective intensity was raised using imageJ standard program feature. In the histogram profile, we categorized pixel intensity ranges from 0–60 for a score value of 3+, 61–120 for 2+, 121–170 for 1+ and 171–230 for 0. We excluded the pixel intensity values above 230 as the abundant fatty tissues in breast tumor tissue sections were found to contribute in the range of 230–255. A macro was developed and plugged in the ImageJ program to receive automated counting of numbers of pixels in the four different intensity zones. After determining these numbers, we applied them to a simple algebraic formula as shown below to determine the score of the image.
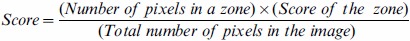



Any image that has more than 66% of pixels in one zone is directly graded without the need to apply the formula. In line with the standard grading procedure, we assigned a 4 tire scoring system i.e. high positive (3+), positive (2+), low positive (1+) and negative (0).

### Statistical AnalysisW

All the statistical analysis was performed using Graph pad and Microsoft Excel software. The significance of difference was obtained by performing the chi square test, and the level was set at P<0.05. For comparison of two grading methods kappa statistical analysis was performed.

## Results

### hNIS Expression is Significantly Different in Major Subtypes of Breast Cancer

**Figure 1:**
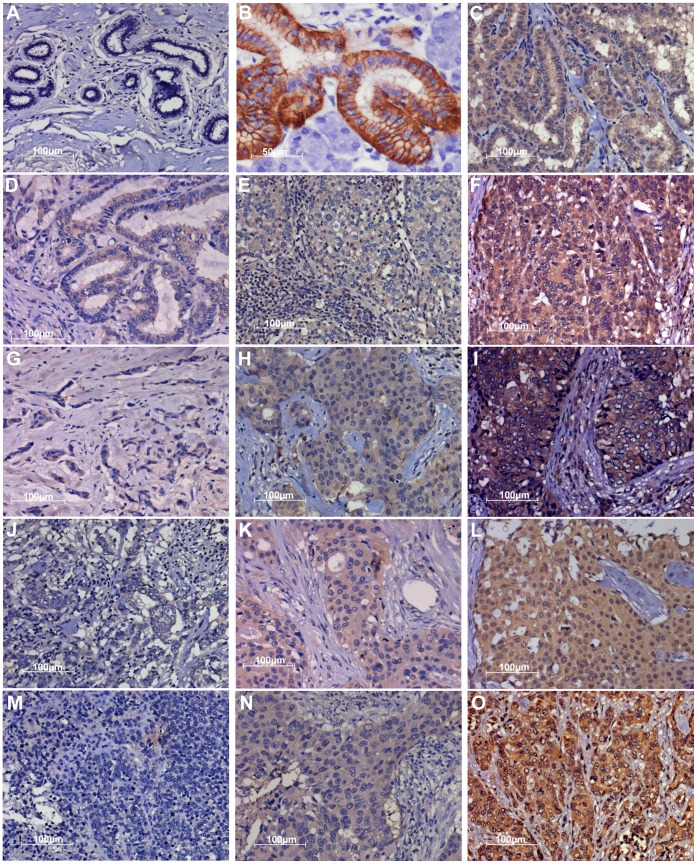
Images of hNIS staining pattern in major subtypes of breast cancer. **A**) Tumor adjacent normal breast tissue showing negative expression of hNIS. **B**) Salivary gland tissue section showing membrane staining for hNIS. **C**) Thyroid papillary carcinoma tissue section as positive control. **D-F**) hNIS expression in ER+ve, PgR+ve, HER2-ve subtype representing 0, 1+ and 2+ score respectively. **G-I**) hNIS expression in ER+ve, PgR+ve, HER2+ve subtype with 0, 1+ and 2+ score respectively. **J-L**) hNIS expression in ER-ve, PgR-ve, HER2+ve subtype with 0, 1+ and 2+ score respectively. **M-O**) hNIS expression in ER-ve, PgR-ve, HER2-ve subtype with 0, 1+ and 2+ scores respectively. All images are captured at 20X magnification.

**Figure 2:**
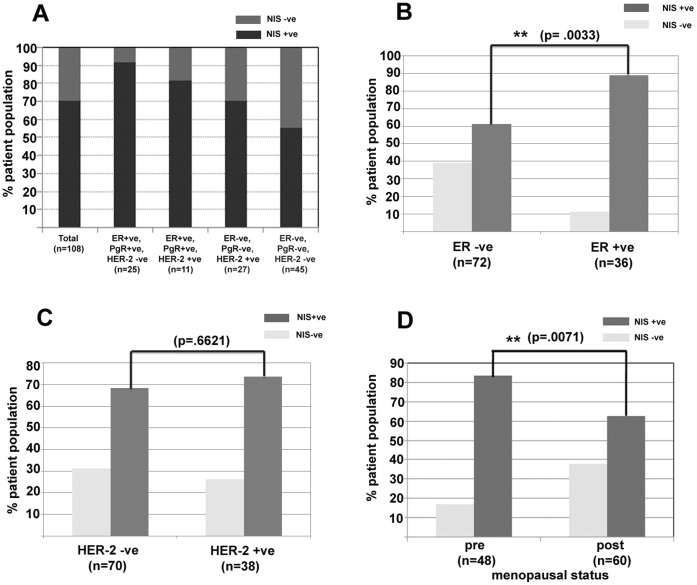
hNIS expression varies in different patient cohorts. **A**) Chart representing positive and negative hNIS expression in 108 cases of breast cancer representing major subtypes. **B**) hNIS positive expression is compared between the ER-ve and ER+ve patient samples indicating significant association of hNIS expression in ER+ve group. **C**) hNIS positive expression is compared between HER2–ve and HER2+ve patient samples indicating no association. **D**) hNIS positive expression in pre- and post-menopausal group of patients indicating significant higher hNIS expression in pre-menopausal women.

The pattern of hNIS staining is mainly diffused cytoplasmic as appeared in representative images shown (**Figure-1)**. Tumor adjacent normal breast tissues are stained negative. Normal salivary gland membrane staining for hNIS expression was evident (Figure-1b). By analyzing 108 patient tumor tissue samples, we found that 70% cases are positively stained **(Figure-2A)**. Subtype specific classification shows that more than 90% samples of ER+ve, PgR +ve, HER2 -ve and 82% of ER+ve, PgR +ve, HER2+ve subtype are positive whereas only 70% HER2 enriched (ER-ve, PgR–ve, HER2+ve) and 56% of ER-ve, PgR–ve, HER2-ve samples are positive for hNIS expression. In comparison to the thyroid papillary case **(Figure-1C)** used as a positive reference for hNIS staining, breast tumor tissues show equivalent or lesser staining intensity and therefore are categorized as medium (2+), low (1+) and negative (0) scores by pathological analysis. None of the breast cancer sample or the thyroid case used as reference shows 3+ scoring. Analyzing the hNIS staining intensity variation among subtypes, we found that out of the 23 positive cases of ER+ve, PgR+ve, HER2-ve subtype, 14 are medium positive and 9 are low positive cases (**Table-S1**). Of the 9 (out of 11) positive ER+ve, PgR+ve, HER2+ve subtype, 4 cases show medium and 5 cases show low intensity staining. In case of HER2 subtype (ER-ve, PgR-ve, HER2+ve), out of 27 cases 7 are medium and 12 low positive cases are recorded. Whereas out of the 45 ER-ve, PgR-ve, HER2-ve subtype cases, only 8 medium, 17 low and 20 negative cases are found. ER+ve, PgR+ve, HER2-ve subtype showed significantly high expression of hNIS as compared to ER-ve, PgR-ve, HER2-ve subtype (p = 0.0014).

### hNIS Expression Positively Correlated with Estrogen Receptor and Premenopausal Status of the Patient

We compared the hNIS positive expression in ER–ve and ER+ve samples. As shown in the **Figure-2B**, out of the 36 ER+ve cases, 32 samples (88.8%) are positive for hNIS expression whereas among the 72 ER-ve cases only 44 cases (61.1%) are positive for hNIS expression. (p = 0.0033). ER+ve cases also show higher intensity staining (2+) in 50% cases whereas in case of ER-ve tumors only 21% samples show 2+ staining intensity. Thus hNIS expression in ER+ve tumors indicates a positive correlation (p = 0.0033) between the two molecules. Further, we also compare hNIS expression with respect to patient HER2 status, preoperative chemotherapy status, and menopausal status. Around 69% HER2–ve tumors show positive hNIS expression whereas 74% HER2+ve tumors show hNIS positivity with a p value of 0.6621 (**Figure-2C**). In case of post-chemotherapy cases 54% tumor samples show positive expression whereas 69% of pre-chemotherapy cases show hNIS positivity (p = 0.0607) (data not shown). In case of post chemotherapy cases hNIS positivity was observed in all 4 ER+ve, PgR+ve, HER2-ve subtype, 1 (out of 2) ER+ve, PgR+ve, HER2+ve subtype, 2 (out of 3) ER-ve, PgR-ve, HER2+ve subtype and 3 (out of 7) ER-ve, PgR-ve, HER2-ve subtype. Therefore, hNIS status does not show any significant association with either HER2 or patient preoperative chemotherapy status.

Based on the reported age in the patient registry, we categorized samples into 2 groups i.e. premenopausal (</ = 50 years) and postmenopausal (>50 years) and compared for hNIS expression. Numbers of samples in these groups are 48 and 60 respectively. In case of pre-menopausal women 81% patients show positive expression of hNIS, whereas 63% of post-menopausal women show hNIS positivity (**Figure-2D**). The hNIS expression in pre-menopausal women is found to be highly significant when compared with the post-menopausal group (p = 0.0071).

### hNIS and HER2 Receptor Expression Overlaps at the Tumor Cell Membrane in Few cases

**Figure 3:**
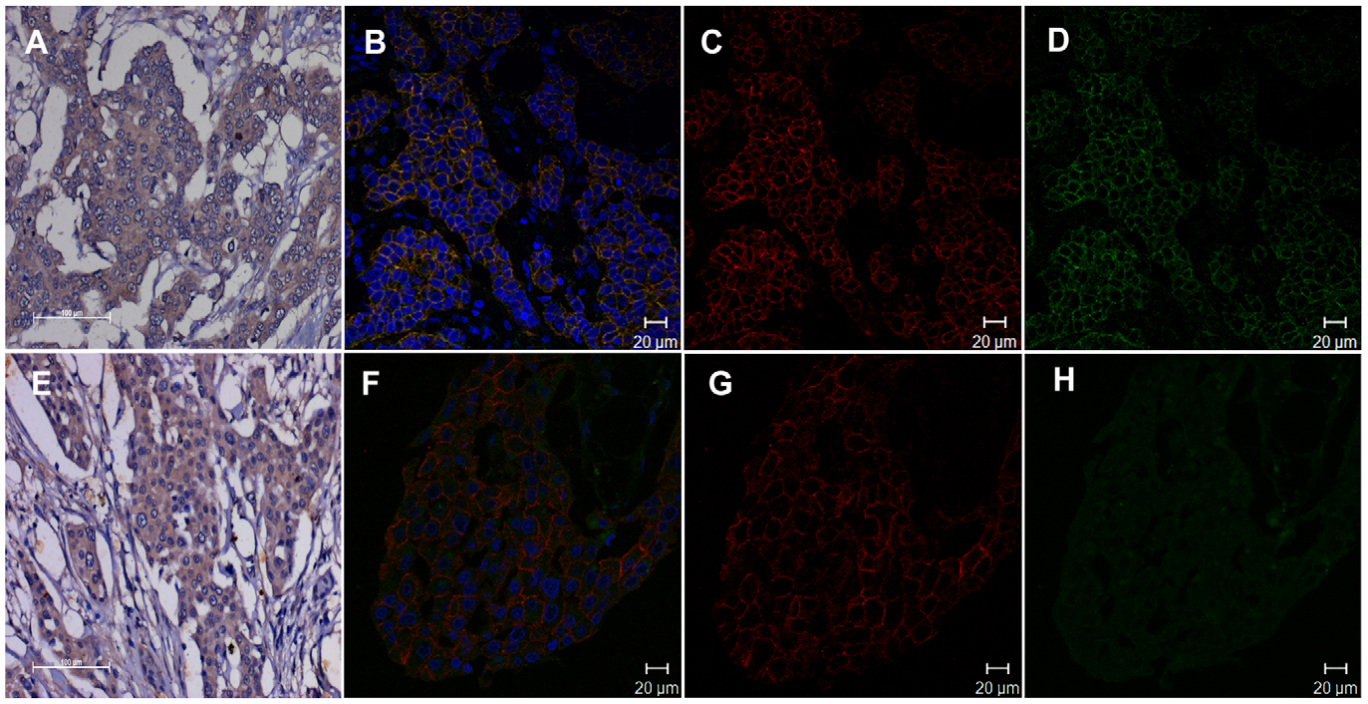
Immunofluorescence staining reveals hNIS and HER2 receptor overlaps at the tumor cell membrane in few cases. Human patient tissue samples stained with hNIS and HER2 specific antibody followed by detection with Alexafluor488 (green) and Dylight633 (red) labeled secondary antibodies respectively. **A, E**) DAB stained tissue sections from two representative patient samples showing similar intracellular expression of hNIS. **B, F**) Dual IF stained confocal overlay images of the same patient samples as in A and E respectively. Upper panel clearly showed good overlapping membrane expression of HER2 and hNIS staining. **C, G**) Corresponding images of HER2 staining in red channel and **D, H**) images of hNIS staining in green channel.

As hNIS is a transporter protein, except for a few reports [5], [6] that have shown distinct membrane staining, mostly it appears as an intracellular protein in breast tumor tissue sections. During this study except for salivary gland section and a negligible number of cases, hNIS appeared as diffused intracellular staining on DAB stained tumor tissue sections (**Figure-1**). This being an important judgment in estimating the utility of hNIS mediated radio-iodine uptake, we attempted to verify that if HER2 (membrane receptor) and hNIS co-localize in the HER2 receptor positive samples. To achieve this, dual IF staining was performed using hNIS and HER2 primary antibodies which were detected by using fluorescent dye labeled secondary antibodies. As represented in **Figure-3**, in a few cases merged confocal microscopic images clearly show co-localization of the two molecules at the cell membrane of tissue section which is not apparent in the image of the same case stained with DAB colorimetric staining method (upper panel). However, as represented in the lower panel of **Figure-3**, in many cases similar membrane aggregation of hNIS protein is absent and the whole cytoplasmic area shows uniform staining in hNIS positive cases by both IF and DAB.

### Digital Image Scoring Method Shows Good Match with Pathological Scoring

**Figure 4:**
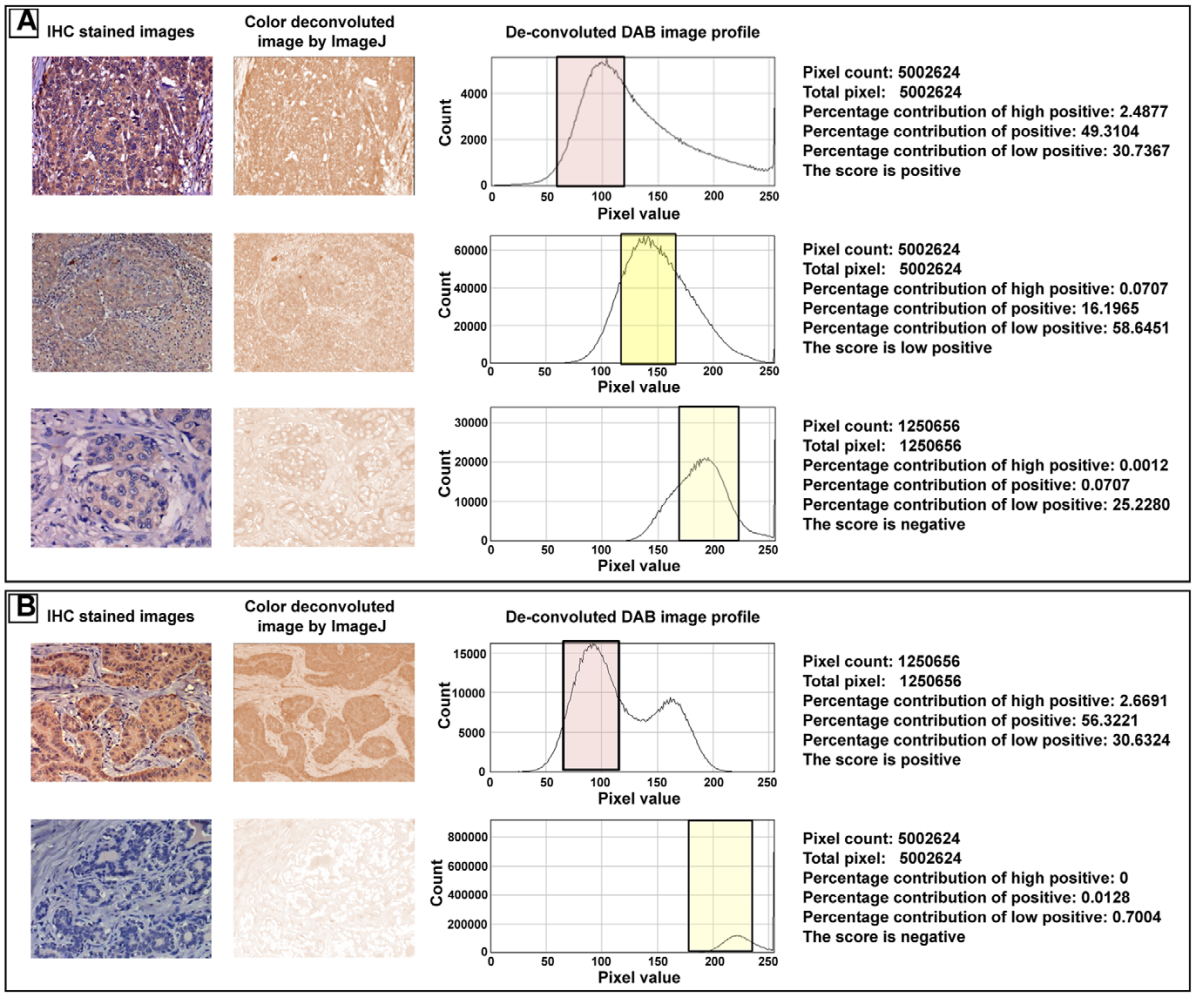
Diagrammatic representation of different zones classified for scoring DAB stained IHC images. **A**) IHC image, their DAB color de-convoluted image, reference histogram profile and pixel analysis data table for each are represented for cases scored as 2+, 1+ and 0. On the histogram profile, subdivision of zones based on pixel intensities are marked with colored boxes as zone 2 (61–120) with pink, zone 3 (121–170) with yellow and zone 4 (171–230) with light yellow. **B**) software-based analysis data set of the papillary thyroid carcinoma sample representing positive (2+) score and tumor adjacent normal sample representing negative score are represented.

**Figure 5:**
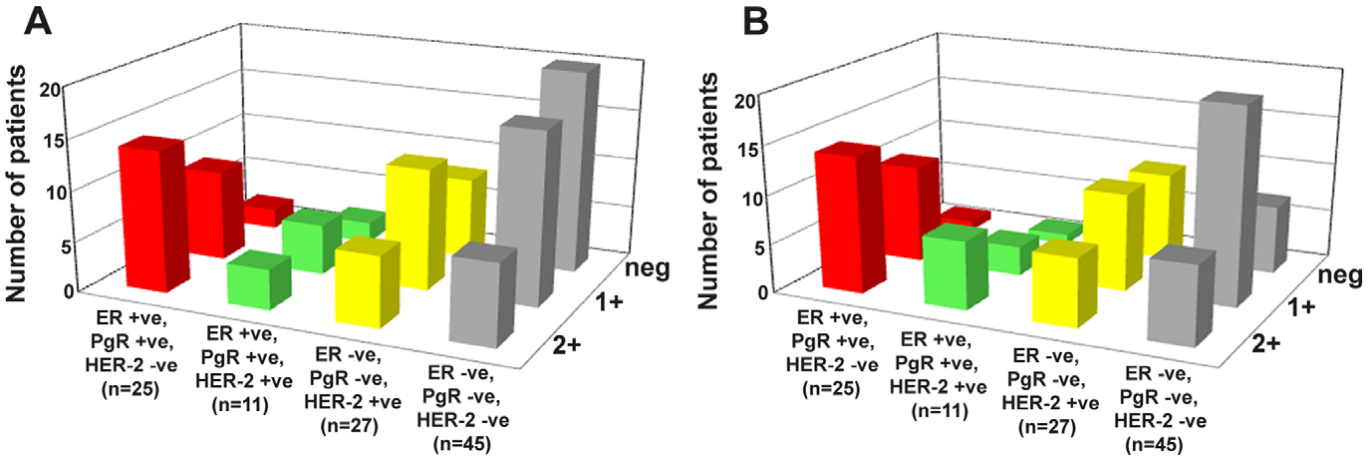
Subtype specific hNIS scoring analysis in breast cancer. A ) Chart representing IHC scoring for hNIS in various breast cancer subtypes by pathological analysis. **B**) IHC scoring for hNIS of the same dataset by semi-automated process developed. On an average 68% match and 75% match with 2+ score is observed between the two scoring methods.

Images of all 108 breast cancer cases were also analyzed by the software developed during this study. The IHC images were first color de-convoluted by modifying the technique described by Ruifrok et al. [26], followed by pixel intensity profiling of de-convoluted DAB stained image using ImageJ program. **Figure-4A** shows representative zones and pixel intensity analysis of IHC images which were scored as 2+, 1+ and 0 respectively by visual estimation. Points to be noted here is that as shown in **Figure-4B**, thyroid papillary tumor case used as a positive control obtained a score of 2+, whereas tumor adjacent normal breast tissue obtained a score of 0. Comparing the scores assigned by pathological analysis with that obtained by the software based process, a 68% match is observed. Out of the 108 cases, 73 cases show similar score by both methods (**Figure-5**). Further, we also found that often software analysis designate lower score in cases where the analyzed image represents low tumor to stroma ratio (**Figure-S1**), requiring physical examination of the whole section. As the data presented above, out of the 35 cases where the analysis differ, 17 cases represent images with low tumor to stroma ratio. Importantly, it is also observed that 75% cases with 2+ score matched by both methods, indicating such digital analysis method can benefit patient stratification for hNIS expression across the laboratories.

## Discussion

Overexpression of hNIS gene in BC is now well established in reports made by several groups across the world. However, the available reports on hNIS expression are often focused at selective groups of patients such as triple negative breast cancer (TNBC) [8]. Because TNBC subgroup lacks alternative targeted treatment options but shows high incidence of hNIS positivity, this group was primarily aimed for hNIS based targeted radio-iodine treatment. Despite this fact, we wanted to conduct a thorough subtype specific analysis across unbiased breast tumor tissue samples. To the best of our knowledge, this is the first comparative IHC assessment of hNIS protein expression in different subtypes of BC, which indicates that significant difference lies among various subtypes. Analyzing over a hundred samples, our result suggests that in an average hNIS protein over-express in 70% of BC cases, where as the previous reports indicated 80–85% positivity overall [5], [7], [8]. Interestingly, our data also show that ER positive cases have much higher incidence of hNIS co-expression i.e. 90% of ER+ve, PgR+ve, HER2 -ve samples and 80% of ER+ve, PgR+ve, HER2+ve samples are found positive for hNIS staining. In converse, only 56% cases of ER-ve, PgR-ve, HER2-ve subtype are found positive which is significantly lower than the earlier reports. Without analyzing cases across the world, it is difficult to estimate at this point if a geographical variation persists in terms of hNIS expression. Coming to the ER and hNIS coexistence in breast tumor tissues, hNIS expression is found to be positive in a significantly high percentage (89%) of ER+ve tumors as oppose to only 61% in ER-ve tumors. Higher numbers of 2+ score for hNIS (equivalent to thyroid control tissue) in ER+ve, PgR+ve subtypes indicate that these subtypes will possibly have good response for hNIS based targeted radio-iodine therapy or radio-iodine probe based PET (Positron Emission Tomography) or SPECT (Single Photon Emission Computed Tomography) diagnosis. Further, significant higher incidence of hNIS expression in pre-menopausal patient group over the post-menopausal patient group perhaps also indicate that estrogen hormone might have a role to play on tumor associated hNIS over-expression. Questioning whether or not ER plays a critical regulatory role on hNIS expression, there are conflicting reports which probably suggests that ER may not play a critical role for hNIS induction in breast tumors and there may be ER-independent mechanisms that promote hNIS gene expression [9], [27], [28]. ER-independent regulation of hNIS over-expression can be further argued with the fact that hNIS is well represented in ER-ve, PgR-ve subtypes as well. However, hNIS do not show any significant relationship with the HER2 status or the preoperative chemotherapy status of the patient. Overall, the study indicates that targeted radio-iodine therapy will provide an alternative option for tumor specific and less toxic treatment in cases where the tumors become resistant to tamoxifen and other chemotherapeutic drugs as well.

Next we envisioned that in order to play its functional role as a transporter, hNIS proteins should be localized at the plasma membrane. But in majority of the cases a diffused intracellular staining pattern is observed. Though a few studies in the past have shown membrane specific staining in breast tumor tissues [5], [6], clinical studies using ^99m^TcO_4_ showed visible tumor by scintigraphic imaging only in 17% of breast cancer patients [20]. This being an important aspect to judge the localization of functional hNIS molecule, reliable determination of immune-reactivity at plasma membrane required further evaluation. Our immune-fluorescence results using HER2 positive cases confirm that in breast tumor tissues differences lie in terms of hNIS membrane staining. In certain cases hNIS staining dominantly overlaps with HER2 receptor staining at the cell surface, whereas in other cases hNIS shows uniform distribution throughout the cytoplasm. Nonetheless, membrane localization of hNIS is not evident in respective cases where DAB staining was performed in parallel. Prior to the translation of hNIS gene based radio-iodine therapy it will be necessary to perform nuclear medicine studies using ^123^I scintigraphic or ^124^I PET imaging in breast cancer patients to determine sufficient radioisotope uptake by hNIS expressing breast cancer tissues.

Taking the study forward, we also report the development of a semi-automated digital image analysis toolbox capable of assigning scores directly by profiling of hNIS staining intensity in IHC digital images. By performing an accuracy comparison study, we see ∼70% match in scoring obtained by manual and software-based method. Considering the biological variations in human tissue samples, unsupervised grading analysis providing such high percentage match with pathological analysis seems to be a good achievement. Further, we also observed that majority of the cases where automated scoring differs by 1 or 2 degree from that of the pathological analysis, are mostly the cases where low tumor to stromal tissue ratio are present (e.g. **Figure-1H, J**) in the images analyzed. The high percentage of pixels in the stroma area representing low intensity/negative values (of <200 pixel value) lowers the average score assigned. One way to overcome this would be to analyze zoomed images taken at higher magnification (**Figure-S1**) or consider averaging the scores obtained from multiple images captured in such cases. Further refinements to improve score assignment by nullifying the averaging effect of pixel intensities within an image area where presence of tumor cell percentage is lower than 50% of the entire field will make it an ideal platform to apply for clinical assessment of hNIS expression.

### Conclusion

In conclusion we report that hNIS protein expresses in 70% cases of breast cancer, but the expression significantly varies among the subtypes of breast cancer. Highest frequency and intensity of hNIS expression is found in ER+ve, PgR+ve, HER2-ve subtype suggesting that hNIS expression is strongly associated with ER expression, whereas it shows no significant association with HER2 and preoperative chemotherapy status of a patient. Co-localization of hNIS with HER2 at the cell membrane is also evident in some cases whereas the protein is mostly present in intracellular location in hNIS positive cases. Further, the semi-automated ImageJ based digital image analysis method developed provides a platform for unbiased, quantitative evaluation of most DAB stained IHC images and serve the purpose of patient stratification across the globe.

## Supporting Information

Figure S1 **Images captured using higher magnification can assign correct scoring by reducing the averaging effect contributed from the stomal areas. A**) Analysis of image score using a 20X image of a case where low tumor cells were present. After color deconvolution, the macro was used on the DAB image to plot a histogram profile and the score was determined as positive. **B)** When an image is captured using a 40X objective focusing on the marked area of the previous image, score was found to be high positive.
(TIF)

Table S1 **Summary of IHC scores for hNIS expression in different subtypes of breast cancer.**
(DOCX)
