# Supplementary figures and images for: Quantitative Immunohistochemical Analysis Reveals Association between Sodium Iodide Symporter and Estrogen Receptor Expression in Breast Cancer
Source: PLoS One. 2013 Jan 14;8(1):e54055. doi: 10.1371/journal.pone.0054055 (PMC3544659; doi:10.1371/journal.pone.0054055)

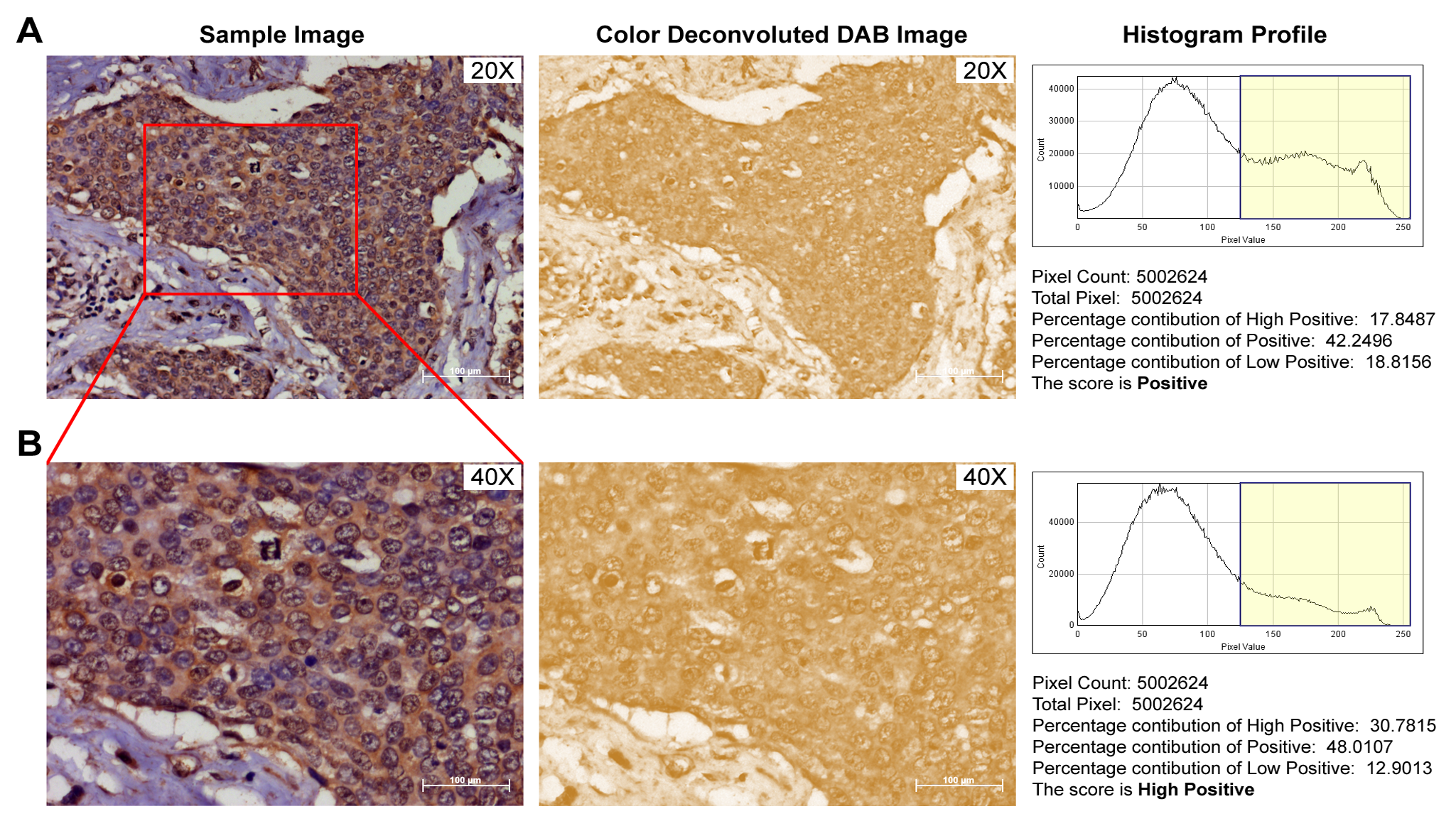

Supplement: Figure S1 — Images captured using higher magnification can assign correct scoring by reducing the averaging effect contributed from the stomal areas. A) Analysis of image score using a 20X image of a case where low tumor cells were present. After color deconvolution, the macro was used on the DAB image to plot a histogram profile and the score was determined as positive. B) When an image is captured using a 40X objective focusing on the marked area of the previous image, score was found to be high positive. (TIF) [file pone.0054055.s001.tif]
