# Supplementary material for: Quantitative Immunohistochemical Analysis Reveals Association between Sodium Iodide Symporter and Estrogen Receptor Expression in Breast Cancer
Source: PLoS One. 2013 Jan 14;8(1):e54055. doi: 10.1371/journal.pone.0054055 (PMC3544659; doi:10.1371/journal.pone.0054055)
Supplement: Table S1 — Summary of IHC scores for hNIS expression in different subtypes of breast cancer. (DOCX) [file pone.0054055.s002.docx]

| Subtypes | Negative (0) score | Low (1+) score | Medium (2+) score |
| --- | --- | --- | --- |
| ER+ve, PgR+ve, HER2-ve (n=25) | 2 | 9 | 14 |
| ER+ve, PgR+ve, HER2+ve (n=11) | 2 | 5 | 4 |
| ER-ve, PgR-ve, HER2 +ve (n=27) | 8 | 12 | 7 |
| ER-ve, PgR-ve, HER2-ve (n=45) | 20 | 17 | 8 |
